# C-Reactive Protein, High-Molecular-Weight Adiponectin and Development of Metabolic Syndrome in the Japanese General Population: A Longitudinal Cohort Study

**DOI:** 10.1371/journal.pone.0073430

**Published:** 2013-09-12

**Authors:** Yoshifumi Saisho, Hiroshi Hirose, Rachel Roberts, Takayuki Abe, Hiroshi Kawabe, Hiroshi Itoh

**Affiliations:** 1 Department of Internal Medicine, Keio University School of Medicine, Tokyo, Japan; 2 Health Center, Keio University School of Medicine, Tokyo, Japan; 3 Center for Clinical Research, Keio University School of Medicine, Tokyo, Japan; University of Tokushima, Japan

## Abstract

**Aims:**

To clarify predictive values of C-reactive protein (CRP) and high-molecular-weight (HMW) adiponectin for development of metabolic syndrome.

**Research Design and Methods:**

We conducted a prospective cohort study of Japanese workers who had participated in an annual health checkup in 2007 and 2011. A total of 750 subjects (558 men and 192 women, age 46±8 years) who had not met the criteria of metabolic syndrome and whose CRP and HMW-adiponectin levels had been measured in 2007 were enrolled in this study. Associations between CRP, HMW-adiponectin and development of metabolic syndrome after 4 years were assessed by logistic regression analysis and their predictive values were compared by receiver operating characteristic analysis.

**Results:**

Among 750 subjects, 61 (8.1%) developed metabolic syndrome defined by modified National Cholesterol Education Program Adult Treatment Panel III (NCEP-ATP III) criteria and 53 (7.1%) developed metabolic syndrome defined by Japan Society for the Study of Obesity (JASSO) in 2011. Although CRP and HMW-adiponectin were both significantly correlated with development of metabolic syndrome, multivariate logistic regression analysis revealed that HMW-adiponectin but not CRP was associated with metabolic syndrome independently of BMI or waist circumference. Adding these biomarkers to BMI or waist circumference did not improve the predictive value for metabolic syndrome.

**Conclusion:**

Our findings indicate that the traditional markers of adiposity such as BMI or waist circumference remain superior markers for predicting metabolic syndrome compared to CRP, HMW-adiponectin, or the combination of both among the Japanese population.

## Introduction

Metabolic syndrome is now widely appreciated as a cluster of metabolic abnormalities such as visceral obesity, hypertension, hyperglycemia and dyslipidemia [1]. To date, incidence of metabolic syndrome is continuously increasing worldwide. Since subjects with metabolic syndrome are at risk for development of type 2 diabetes, cardiovascular disease (CVD) and cancer [1], [2], prevention of metabolic syndrome is an urgent issue.

Higher levels of C-reactive protein (CRP) and lower levels of high-molecular-weight (HMW) adiponectin have been both reported to correlate with obesity and metabolic syndrome [3], [4], [5], [6]. These biomarkers have been also reported to correlate with type 2 diabetes and CVD [5], [7], [8], [9], [10], [11]. Therefore, these biomarkers appear to be useful to detect subjects at high risk of metabolic syndrome, type 2 diabetes and CVD.

Although associations between CRP or HMW-adiponectin and metabolic syndrome have been well established, there have been few studies to examine the usefulness of combination of both biomarkers for prediction of metabolic syndrome. Especially, in clinical settings it is essential to compare the predictive utility of these biomarkers for metabolic syndrome with that of the traditional markers. Therefore, in this longitudinal cohort study we sought to address the following questions: 1) Is there any additive effect of the combination of CRP and HMW-adiponectin for prediction of metabolic syndrome compared with each of them alone? 2) Are predictive values of the traditional markers such as BMI or waist circumference for metabolic syndrome improved by adding these biomarkers?

## Methods

### Ethics Statement

The present study was conducted according to the principles expressed in the Declaration of Helsinki. Written informed consent was obtained from each subject after a full explanation of the purpose, nature and risk of all procedures used. The protocol was approved by the ethical review committees of the Health Center and the Faculty of Medicine, Keio University School of Medicine, Tokyo, Japan.

### Subjects

We conducted a prospective cohort study of 1,552 Japanese teachers and workers at Keio University (1,067 men and 485 women) who had participated in an annual health checkup in 2007. More than 95% of the workers and teachers at Keio University participated in this annual health checkup. In this study, we primarily enrolled subjects aged 40 years and older because the incidence of metabolic syndrome is low in the subjects younger than 40 years old [12]. Then we randomly enrolled subjects younger than 40 years old. As a result, we measured CRP and HMW-adiponectin in 1,250 subjects out of 1,552 subjects (81%). Among them, 42 subjects were excluded because of their CRP>0.5 mg/L. Of those (1,208 subjects), 884 subjects (73%, 676 men and 208 women) had been followed up in an annual health checkup in 2011. Finally, a total of 750 subjects (558 men and 192 women, age 46±8 years) who had not met the criteria of metabolic syndrome defined by National Cholesterol Education Program Adult Treatment Panel III (NCEP-ATP III) and Japan Society for the Study of Obesity (JASSO) in 2007 were enrolled in this study (Table 1). The subjects with whom we were not able to follow up in 2011 (N = 324) were older (49±14 vs. 47±8 years, p<0.05) and predominantly female (32.4% vs. 23.5%, p<0.05) than the study participants (N = 884), although the difference was clinically small, presumably reflecting retirement or resignation from work. Information regarding smoking, alcohol intake, physical activity and medication was obtained from questionnaires to the subjects.

**Table 1:** Characteristics of subjects according to development of metabolic syndrome after 4 years.

	Total	MetS		JMetS	
		(−)	(+)	(−)	(+)
N	750	689	61	697	53
Male (%)	74.4	73.6	83.6	72.9	94.3†
Age (years)	46±8	46±8	49±8**	46±8	50±7†
Height (m)	1.68±0.08	1.68±0.08	1.69±0.08	1.68±0.08	1.69±0.06
Weight (kg)	63.4±15.5	62.7±10.5	71.2±10.6†	62.7±10.6	71.4±8.8†
BMI (kg/m^2^)	22.4±2.9	22.2±2.7	24.9±3.0†	22.2±2.8	24.9±2.8†
Waist circumference (cm)	80.1±8.2	79.5±8.0	86.7±7.7†	79.6±8.0	87.5±6.6†
Systolic blood pressure (mmHg)	120±16	119±15	132±16†	119±15	132±14†
Diastolic blood pressure (mmHg)	75±11	75±11	84±11†	75±11	84±8†
Heart rate (bpm)	76±12	75±12	79±11*	75±12	79±11*
Current smoking (%)	8.9	9.1	6.6	8.8	11.3
Alcohol intake (≥20 g/day) (%)	21.3	20.6	29.5	19.8	41.5**
No exercise (<150 min/week) (%)	61.9	62.3	57.4	62.4	54.7
Antihypertensives (%)	6.4	4.8	24.6†	4.6	30.2†
Lipid-lowering agents (%)	1.7	1.2	8.2**	1.9	0.0
Oral hypoglycemic agents (%)	0.7	0.7	0.0	0.4	3.8*
AST (IU/L)	20 (17–24)	20 (17–24)	20 (17–25)	20 (17–23)	24 (19–28)**
ALT (IU/L)	20 (14–26)	19 (14–26)	24 (17–36)*	19 (14–26)	26 (20–36)**
γ-GTP (IU/L)	26 (17–42)	25 (17–40)	37 (26–71)*	24 (17–40)	47 (32–80)**
ALP (IU/L)	198±59	198±58	206±62	198±59	201±58
Glucose (mg/dL)	89 (85–94)	89 (85–94)	94 (89–97)*	89 (85–94)	94 (91–98)**
Total cholesterol (mg/dL)	206±30	205±29	216±35*	206±30	207±30
LDL-cholesterol (mg/dL)	121±28	120±27	135±35**	121±27	123±32
HDL-cholesterol (mg/dL)	64±16	64±16	56±12†	64±16	55±11†
Triglyceride (mg/dL)	79 (53–113)	76 (52–110)	114 (84–148)†	75 (52–109)	123 (97–149)†
Uric acid (mg/dL)	5.6±1.4	5.6±1.4	6.3±1.1†	5.6±1.3	6.6±1.2†
Creatinine (mg/dL)	0.78±0.14	0.78±0.14	0.81±0.16	0.78±0.14	0.84±0.13**
Insulin (mU/L)	4.1 (3.0–5.8)	4.1 (3.0–5.7)	5.2 (3.9–7.1)	4.1 (3–5.6)	5.8 (3.9–7.4)*
HOMA-IR	0.91 (0.67–1.31)	0.89 (0.66–1.27)	1.18 (0.86–1.68)*	0.89 (0.66–1.26)	1.40 (0.94–1.75)**
CRP (mg/L)	0.28 (0.13–0.62)	0.26 (0.12–0.60)	0.54 (0.23–0.78)†	0.26 (0.12–0.6)	0.54 (0.33–0.74)†
HMW-adiponectin (µg/mL)	4.0 (2.5–6.3)	4.2 (2.6–6.5)	2.7 (1.8–3.9)†	4.3 (2.6–6.5)	2.3 (1.6–3.3)†
C/A ratio	0.08 (0.03–0.19)	0.07 (0.02–0.18)	0.20 (0.08–0.41)†	0.07 (0.02–0.18)	0.24 (0.10–0.41)†

Values are expressed as mean ± SD for variables with normal distribution and median (interquartile range) for those with non-normal distribution. MetS; metabolic syndrome, JMetS; Japanese metabolic syndrome.

* P<0.05,

** P<0.01,

† P<0.001 vs. MetS(−) or JMetS(−).

### Measurement

Systolic and diastolic blood pressure was measured in the sitting position after resting for at least 3 min using an automatic electronic sphygmomanometer (BP-103i II; Nippon Colin, Komaki, Japan) with cuff size of 14.5 cm in width and 52.0 cm in length. Blood samples were collected in the morning after an overnight fast.

Plasma glucose and serum lipids were assayed by routine automated laboratory methods as previously described [13]. Serum insulin concentration was measured by an enzyme immunoassay, using a commercially available kit (Tosoh, Tokyo, Japan), with intra- and interassay coefficients of 2.9 to 4.6% and 4.5 to 7.0%, respectively. The insulin resistance index was assessed by a homeostasis model assessment of insulin resistance (HOMA-IR), which was calculated as fasting serum insulin (mU/L) × fasting plasma glucose (mmol/L)/22.5 [14].

HMW-adiponectin was measured using a commercially available kit (HMW adiponectin ELISA Kit, Fujirebio, Tokyo, Japan) as previously reported [13]. This ELISA system does not need a denaturing step, and the monoclonal antibody (IH7) is reported to react specifically with the HMW form of adiponectin [15]. The dilution curve was parallel to the standard curve. Intra- and interassay coefficients were 2.4 to 3.0% and 4.2 to 5.1%, respectively. We have previously reported that HMW-adiponectin and HMW-adioponectin to total adiponectin ratio were more sensitively associated with metabolic syndrome than total adiponectin alone [16], [17], [18]. Thus we measured HMW-adiponectin rather than total adiponectin in this study. Serum CRP levels were measured by nephelometry, a latex particle-enhanced immunoassay (N Latex CRP II, Dade Behring, Tokyo, Japan) with both intra- and interassay coefficients of <5.0%. For the analysis of CRP values under the assay detection limit of 0.04 mg/L, an approximate value of 0.02 mg/L was used.

### Definition of Metabolic Syndrome

Metabolic syndrome (MetS) was defined according to the revised NCEP ATP III criteria [1] as 3 or more of 5 components in which the cut-off point of waist circumference was modified for Japanese as ≥90 cm in men and ≥80 cm in women according to the recommendation by the International Diabetes Federation (IDF) [19], the cut-off points of the other components were systolic blood pressure ≥130 mmHg and/or diastolic blood pressure ≥85 mmHg for blood pressure, ≥150 mg/dL for triglycerides, <40 mg/dL in men and <50 mg/dL in women for HDL-cholesterol, and ≥100 mg/dL for fasting plasma glucose. Subjects receiving antihypertensive, lipid-lowering agent or hypoglycemic medication were considered to have the respective component. Japanese metabolic syndrome (JMetS) defined by the Examination Committee for Criteria of Metabolic Syndrome [20] was also examined. The criteria of JMetS is waist circumference ≥85 cm in men and ≥90 cm in women plus 2 or more of the following 3 components; systolic blood pressure ≥130 mmHg and/or diastolic blood pressure ≥85 mmHg, triglycerides ≥150 mg/dL and/or HDL cholesterol <40 mg/dL, and fasting plasma glucose ≥110 mg/dL.

### Statistical Analysis

Comparisons between the two groups were performed with Student’s t-tests or Fisher’s exact tests and odds ratios were determined by logistic regression analysis using the Statistical Package for the Social Sciences (version 19.0; SPSS, Chicago, IL, USA). Receiver operating characteristic (ROC) curves for MetS and JMetS were plotted separately and the area under the curve (AUC) (also referred to as C-statistic in the case of a binary outcome) of ROC curves was calculated. ROC analysis was performed using Proc Logistic in SAS 9.2 (SAS Institute, Cary, NC), which estimates AUC using the trapezoidal method of integration of the sensitivity curve. All normally distributed data are expressed as mean ± S.D., while non-normal data are expressed as median (interquartile range) and the logarithms of the non-normal data were used for the analyses. Values of p<0.05 were considered statistically significant.

## Results

### Baseline Characteristics of Subjects According to Development of Metabolic Syndrome

During 4 years, 61 subjects (8.1%, 51 men and 10 women) developed MetS and 53 (7.1%, 50 men and 3 women) developed JMetS, respectively. Comparisons of baseline characteristics according to the development of metabolic syndrome are shown in Table 1. CRP and C/A ratio were significantly higher, and HMW-adiponectin was significantly lower in subjects who developed metabolic syndrome compared with those who did not (all p<0.001). HMW-adiponectin and CRP are significantly correlated with BMI and waist circumference (Table 2), whereas the correlation was stronger in HMW-adiponectin compared with CRP.

**Table 2:** Correlations among HMW-adiponectin, CRP, BMI and waist circumference.

	BMI	Waist circumference	HMW-adiponectin	CRP
BMI	1	0.853**	−0.413**	0.194**
Waist circumference	0.853**	1	−0.409**	0.221**
HMW-adiponectin	−0.413**	−0.409**	1	−0.117*
CRP	0.194**	0.221**	−0.117*	1

* P = 0.001,

** P<0.001.

### Association between CRP, HMW-adiponectin and Development of Metabolic Syndrome

In univariate logistic regression analysis, both CRP and HMW-adiponectin were significantly associated with development of metabolic syndrome (odds ratio: 1.54 and 0.45 for MetS and 1.65 and 0.33 for JMetS, respectively, Table 3). However, when the model was adjusted for age, sex and traditional markers of adiposity such as BMI or waist circumference, HMW-adiponectin, but not CRP, was significantly associated with development of metabolic syndrome (Table 3). Adding the other variables which relate to development of metabolic syndrome to the model did not change the results (Models 3 and 4, Table S1).

**Table 3:** Odds ratios (95% CI) according to univariate and multivariate logistic regression analyses of HMW-adiponectin and CRP for development of metabolic syndrome.

	MetS			JMetS		
	Univariate	Multivariate		Univariate	Multivariate	
Variable		Model 1	Model 2		Model 1	Model 2
Age (years)	1.05 (1.01–1.09)**	1.04 (1.00–1.08)*	1.03 (0.99–1.07)	1.09 (1.04–1.13)†	1.07 (1.03–1.12)**	1.07 (1.02–1.11)**
Sex (male = 1, female = 0)	1.83 (0.91–3.68)	0.55 (0.24–1.28)	0.60 (0.26–1.36)	6.20 (1.91–20.11)**	0.56 (0.16–1.98)	0.53 (0.15–1.88)
Ln{CRP (mg/L)}	1.54 (1.22–1.96)†	1.21 (0.92–1.59)	1.21 (0.92–1.59)	1.65 (1.28–2.13)†	1.29 (0.95–1.73)	1.26 (0.93–1.71)
Ln{HMW-adiponectin (µg/mL)}	0.45 (0.31–0.64)†	0.60 (0.38–0.96)*	0.56 (0.36–0.88)*	0.33 (0.22–0.49)†	0.50 (0.30–0.81)**	0.48 (0.30–0.78)**
BMI	1.37 (1.25–1.51)†	1.31 (1.18–1.46)†	–	1.36 (1.23–1.50)†	1.25 (1.12–1.39)†	–
Waist circumference (cm)	1.11 (1.07–1.15)†	–	1.09 (1.05–1.13)†	1.13 (1.09–1.17)†	–	1.09 (1.05–1.14)†

CI; confidence interval.

* P<0.05,

** P<0.01,

† P<0.001.

### Predictive Values of CRP and HMW-adiponectin for Metabolic Syndrome

Finally we evaluated additive effects of CRP and HMW-adiponectin on BMI and waist circumference for prediction of metabolic syndrome (Table 4). ROC analysis revealed that there is no significant change in the AUC of the combination of CRP and HMW-adiponectin compared with that of each of them alone. The AUC of BMI or waist circumference itself was greater than that of CRP, HMW-adiponectin or the combination of both. Comparison between predictive ability of the models using CRP and HMW-adiponectin after adjusting for BMI or waist circumference, and the models using BMI or waist circumference alone, was done using the ROCCONTRAST statement in SAS. All but one of these comparisons showed no significant difference, but the only significant difference was not meaningful, as the actual difference in AUC was less than 1%. Thus the AUC of BMI or waist circumference was not significantly improved by adding CRP, HMW-adiponectin or both. These findings did not change when the subjects were stratified by sex (Tables S2 and S3).

**Table 4:** Comparison of predictive values of biomarkers for metabolic syndrome.

Variables	AUC of ROC curve (95% CI)	
	MetS	JMetS
CRP (mg/L)	0.646 (0.581–0.711)	0.683 (0.626–0.741)
HMW-adiponectin(ADPN) (µg/mL)	0.674 (0.610–0.738)	0.735 (0.671–0.799)
C/A ratio	0.698 (0.637–0.759)	0.753 (0.699–0.807)
ADPN+CRP	0.690 (0.627–0.752)	0.737 (0.673–0.800)
BMI (kg/m^2^)	0.763 (0.711–0.816)	0.758 (0.705–0.811)
Waist circumference(WC) (cm)	0.794 (0.751–0.836)	0.754 (0.703–0.806)
BMI+WC	0.795 (0.751–0.840)	0.763 (0.712–0.814)
BMI+CRP	0.764 (0.712–0.817)	0.761 (0.708–0.813)
BMI+ADPN	0.795 (0.742–0.849)	0.769 (0.717–0.822)
BMI+C/A ratio	0.770 (0.719–0.822)	0.762 (0.710–0.814)
BMI+ADPN+CRP	0.795 (0.742–0.849)	0.772 (0.719–0.824)
WC+CRP	0.754 (0.702–0.806)	0.797 (0.755–0.839)
WC+ADPN	0.768 (0.713–0.822)	0.810 (0.761–0.859)
WC+C/A ratio	0.758 (0.707–0.810)	0.800 (0.759–0.842)
WC+ADPN+CRP	0.768 (0.714–0.823)	0.810 (0.761–0.859)

AUC; area under the curve, ROC; receiver operating characteristics, CI; confidence interval, C/A ratio; CRP to HMW-adiponectin ratio. Lines with variables written with “+” signs indicate that the ROC given is a measure of how well the combination of variables listed explain MetS or JMetS, where values closer to 1 indicate better explanation.

## Discussion

In this study, we report that 1) Combination of CRP and HMW-adiponectin did not improve predictive value for metabolic syndrome compared with each of them alone, 2) Adding these biomarkers to BMI or waist circumference failed to improve predictive value for metabolic syndrome among the Japanese population.

CRP and HMW-adiponectin are both well-known markers for metabolic syndrome, type 2 diabetes and CVD in various ethnics including Japanese [3], [4], [5], [6], [7], [8], [9], [10], [11]. In this study, we also showed that higher CRP and lower HMW-adiponectin were associated with future development of metabolic syndrome defined by either modified NCEP criteria or JASSO criteria. However, the association between metabolic syndrome and CRP, but not HMW-adiponectin, was markedly attenuated after adjustment for age, sex and BMI or waist circumference. It has been reported that CRP is more closely correlated with obesity than metabolic syndrome [21], [22], [23]. Our findings also suggested that the association between CRP and metabolic syndrome are largely explained by obesity.

On the other hand, the association between HMW-adiponectin and metabolic syndrome was significant independently of BMI or waist circumference. Adiponectin is an adipokine secreted from adipose tissue and negatively correlated with visceral obesity and presence of CVD [5], [6], [8], [9], [10], [11]. In animal studies adiponectin has been shown to ameliorate metabolic parameters and suppress progression of atherosclerosis [24]. Specifically, we have shown, as well as others in the field that HMW-adiponectin is a more sensitive marker for metabolic syndrome [16], [17], [18], [25] and type 2 diabetes [26], [27] than total adiponectin. However, the usefulness of HMW-adiponectin for prediction of metabolic syndrome compared with the traditional markers remains uncertain. In this study, the predictive value of HMW-adiponectin for metabolic syndrome by ROC analysis was not significantly greater than that of BMI or waist circumference and adding HMW-adiponectin to BMI or waist circumference did not improve predictive ability, suggesting the lack of utility of HMW-adiponectin to predict future development of metabolic syndrome.

In this study, we further investigated the utility of combination of CRP and HMW-adiponectin for predicting metabolic syndrome. Tabara et al. have reported the synergistic effect of CRP and HMW-adiponectin for prediction of metabolic syndrome in a general population [28]. On the other hand, we and others have previously reported that C/A ratio did not improve the predictive ability for metabolic syndrome compared with each of them alone [13], [29]. Recently, Ong et al. have reported that CRP and total adiponectin levels independently predict the deterioration of glycemia [30]. However, to our knowledge, there has been no study to investigate the utility of combination of both markers for predicting future development of metabolic syndrome in a longitudinal cohort. In the present study, we demonstrated that there is little additive effect of combination of CRP and HMW-adiponectin or C/A ratio for predicting metabolic syndrome compared with each of them alone.

Finally, we examined the additive effect of these biomarkers on the traditional markers for prediction of metabolic syndrome. As a result, ROC analysis revealed that adding CRP and HMW-adiponectin to the traditional markers did not improve the predictive ability for metabolic syndrome. Recently, it has been reported that inflammatory biomarkers including CRP failed to predict type 2 diabetes after adjustment for the traditional markers such as age, sex, BMI and waist circumference [31], [32], whereas Ong et al., have reported that adding CRP and adiponectin to the traditional markers improved predictive ability for deterioration in glycemia, especially in women [30]. Further studies are needed to clarify whether the combination of CRP and HMW-adiponectin improve predictive ability for development of metabolic syndrome and type 2 diabetes.

There are limitations in this study. The relatively small number of subjects who developed metabolic syndrome in this study might reduce the ability to detect a statistical difference among the parameters. However, the high follow-up rate of this study (73%) suggested that the effect of selection bias was low. Second, the proportion of women in this study was relatively small and there were only 10 and 3 women who developed MetS and JMets respectively. Thus our findings in women should be confirmed in future studies with a larger sample size, although we found consistent results when we conducted subanalyses of men and women separately. Third, since the original criteria defined by JASSO is used for the diagnosis of metabolic syndrome in Japan, our findings may not apply to other countries or ethnicities which use different criteria of metabolic syndrome. However, we confirmed the findings by use of two different definitions of metabolic syndrome in this study. As we focused to the development of metabolic syndrome in this study, we were not able to exclude the possibility that CRP and HMW-adiponectin may be useful to predict each component of metabolic syndrome such as impaired glucose tolerance and dyslipidemia. Finally, since our study population was limited to healthy middle-aged Japanese (i.e., teachers and workers at University), the results of this study may not be applied to other population such as children and adolescents, and particularly elderly in which the higher adiponectin levels have been shown to associate with higher risk of CVD and mortality [33], [34], [35], [36].

In conclusion, in this study we reported that CRP, HMW-adiponectin or the combination of both did not improve the predictive value of BMI and waist circumference for metabolic syndrome. Our findings indicate that the traditional markers of adiposity such as BMI or waist circumference are still superior markers for predicting metabolic syndrome among the Japanese population.

## Supporting Information

Table S1 **Odds ratios (95% CI) according to multivariate logistic regression analyses of HMW-adiponectin and CRP for development of metabolic syndrome.**
(DOC)

Table S2 **Comparison of predictive values of biomarkers for metabolic syndrome in men.**
(DOC)

Table S3 **Comparison of predictive values of biomarkers for metabolic syndrome in women.**
(DOC)
