# Supplementary material for: C-Reactive Protein, High-Molecular-Weight Adiponectin and Development of Metabolic Syndrome in the Japanese General Population: A Longitudinal Cohort Study
Source: PLoS One. 2013 Sep 12;8(9):e73430. doi: 10.1371/journal.pone.0073430 (PMC3772031; doi:10.1371/journal.pone.0073430)
Supplement: Table S1 — Odds ratios (95% CI) according to multivariate logistic regression analyses of HMW-adiponectin and CRP for development of metabolic syndrome. (DOC) [file pone.0073430.s001.doc]

Table S1. Odds ratios (95% CI) according to multivariate logistic regression analyses of HMW-adiponectin and CRP for development of metabolic syndrome.

|  | MetS | | JMetS | |
| --- | --- | --- | --- | --- |
| Variables | Model 3 | Model 4 | Model 3 | Model 4 |
| Age (years) | 1.03 (0.99-1.07) | 1.02 (0.98-1.06) | 1.06 (1.02-1.11)** | 1.05 (1.01-1.10)* |
| Sex (male=1, female=0) | 0.39 (0.15-1.01) | 0.38 (0.15-0.99)* | 0.93 (0.24-3.61) | 0.99 (0.26-3.86) |
| Ln{CRP (mg/L)} | 1.25 (0.94-1.66) | 1.25 (0.94-1.67) | 1.32 (0.96-1.82) | 1.29 (0.94-1.78) |
| Ln{HMW-adiponectin (μg/mL)} | 0.67 (0.41-1.10) | 0.65 (0.40-1.06) | 0.50 (0.30-0.85)* | 0.49 (0.29-0.82)** |
| BMI | 1.22 (1.01-1.47)** | - | 1.22 (1.08-1.38)** | - |
| Waist circumference (cm) | - | 1.07 (1.03-1.12)† | - | 1.09 (1.04-1.14)† |
| Smoking status (yes = 1, no = 0) | 0.68 (0.22-2.11) | 0.61 (0.20-1.88) | 0.76 (0.26-2.19) | 068 (0.23-1.99) |
| Alcohol intake (≥20g/day) (yes =1, no = 0) | 1.54 (0.79-3.03) | 1.60 (0.83-3.12) | 1.92 (0.96-3.84) | 1.97 (0.99-3.93) |
| No exercise (<150 min/week) (yes = 1, no = 0) | 1.00 (0.56-1.81) | 0.98 (0.54-1.76) | 1.08 (0.56-2.06) | 1.07 (0.56-2.05) |
| Ln{γ-GTP (IU/L)} | 1.00 (0.99-1.01) | 1.00 (0.99-1.01) | 1.00 (1.00-1.01) | 1.00 (1.00-1.01) |
| LDL-cholesterol (mg/dL) | 1.01 (1.00-1.02)* | 1.01 (1.00-1.02)* | 0.99 (0.98-1.01) | 0.99 (0.98-1.00) |
| Uric acid (mg/dL) | 1.26 (0.95-1.67) | 1.28 (0.97-1.70) | 1.38 (1.02-1.87)* | 1.37 (1.01-1.85)* |

CI; confidence interval. * P <0.05, ** P <0.01, † P <0.001.
