# Supplementary material for: C-Reactive Protein, High-Molecular-Weight Adiponectin and Development of Metabolic Syndrome in the Japanese General Population: A Longitudinal Cohort Study
Source: PLoS One. 2013 Sep 12;8(9):e73430. doi: 10.1371/journal.pone.0073430 (PMC3772031; doi:10.1371/journal.pone.0073430)
Supplement: Table S2 — Comparison of predictive values of biomarkers for metabolic syndrome in men. (DOC) [file pone.0073430.s002.doc]

Table S2. Comparison of predictive values of biomarkers for metabolic syndrome in men.

| Variables | AUC of ROC curve (95% CI) | |
| --- | --- | --- |
|  | MetS | JMetS |
| CRP (mg/L) | 0.597 (0.521-0.673) | 0.627 (0.562-0.693) |
| HMW-adiponectin (ADPN) (μg/mL) | 0.655 (0.583-0.727) | 0.677 (0.601-0.752) |
| C/A ratio | 0.656 (0.584-0.728) | 0.686 (0.620-0.752) |
| ADPN + CRP | 0.664 (0.594-0.734) | 0.678 (0.602-0.753) |
| BMI (kg/m^2^) | 0.711 (0.643-0.778) | 0.699 (0.636-0.762) |
| Waist circumference (WC) (cm) | 0.728 (0.667-0.788) | 0.742 (0.689-0.794) |
| BMI + WC | 0.728 (0.665-0.790) | 0.742 (0.689-0.795) |
| BMI + CRP | 0.715 (0.648-0.783) | 0.700 (0.637-0.763) |
| BMI + ADPN | 0.738 (0.674-0.802) | 0.724 (0.658-0.790) |
| BMI + C/A ratio | 0.718 (0.652-0.784) | 0.706 (0.643-0.768) |
| BMI + ADPN + CRP | 0.744 (0.680-0.807) | 0.725 (0.658-0.791) |
| WC + CRP | 0.726 (0.664-0.787) | 0.738 (0.685-0.791) |
| WC + ADPN | 0.751 (0.688-0.813) | 0.754 (0.694-0.814) |
| WC + C/A ratio | 0.732 (0.672-0.792) | 0.749 (0.698-0.801) |
| WC + ADPN + CRP | 0.752 (0.689-0.815) | 0.754 (0.694-0.814) |

AUC; area under the curve, ROC; receiver operating characteristics, CI; confidence interval, C/A ratio; CRP to HMW-adiponectin ratio. Lines with variables written with “+” signs indicate that the ROC given is a measure of how well the combination of variables listed explain MetS or JMetS, where values closer to 1 indicate better explanation.
