# Supplementary material for: C-Reactive Protein, High-Molecular-Weight Adiponectin and Development of Metabolic Syndrome in the Japanese General Population: A Longitudinal Cohort Study
Source: PLoS One. 2013 Sep 12;8(9):e73430. doi: 10.1371/journal.pone.0073430 (PMC3772031; doi:10.1371/journal.pone.0073430)
Supplement: Table S3 — Comparison of predictive values of biomarkers for metabolic syndrome in women. (DOC) [file pone.0073430.s003.doc]

Table S3. Comparison of predictive values of biomarkers for metabolic syndrome in women.

| Variables | AUC of ROC curve (95% CI) | |
| --- | --- | --- |
|  | MetS | JMetS |
| CRP (mg/L) | 0.770 (0.626-0.913) | 0.931 (0.858-1.000) |
| HMW-adiponectin (ADPN) (μg/mL) | 0.710 (0.576-0.844) | 0.913 (0.811-1.000) |
| C/A ratio | 0.784 (0.637-0.931) | 0.979 (0.955-1.000) |
| ADPN + CRP | 0.745 (0.594-0.896) | 0.956 (0.896-1.000) |
| BMI (kg/m^2^) | 0.903 (0.846-0.960) | 0.995 (0.984-1.000) |
| Waist circumference (WC) (cm) | 0.842 (0.758-0.926) | 0.969 (0.943-0.995) |
| BMI + WC | 0.904 (0.847-0.960) | 0.995 (0.984-1.000) |
| BMI + CRP | 0.899 (0.837-0.960) | 0.997 (0.988-1.000) |
| BMI + ADPN | 0.891 (0.823-0.958) | 0.997 (0.989-1.000) |
| BMI + C/A ratio | 0.902 (0.845-0.960) | 0.997 (0.988-1.000) |
| BMI + ADPN + CRP | 0.892 (0.821-0.962) | 1.000 (1.000-1.000) |
| WC + CRP | 0.838 (0.752-0.924) | 0.968 (0.942-0.994) |
| WC + ADPN | 0.855 (0.773-0.937) | 0.977 (0.940-1.000) |
| WC + C/A ratio | 0.849 (0.766-0.932) | 0.975 (0.949-1.000) |
| WC + ADPN + CRP | 0.852 (0.769-0.935) | 0.979 (0.936-1.000) |

AUC; area under the curve, ROC; receiver operating characteristics, CI; confidence interval, C/A ratio; CRP to HMW-adiponectin ratio. Lines with variables written with “+” signs indicate that the ROC given is a measure of how well the combination of variables listed explain MetS or JMetS, where values closer to 1 indicate better explanation.
